# Supplementary figures and images for: Generation of Novel Tumour-Selective SEA Superantigen-Based Peptides with Improved Safety and Efficacy for Precision Cancer Immunotherapy
Source: Int J Mol Sci. 2024 Aug 30;25(17):9423. doi: 10.3390/ijms25179423 (PMC11395200; doi:10.3390/ijms25179423)

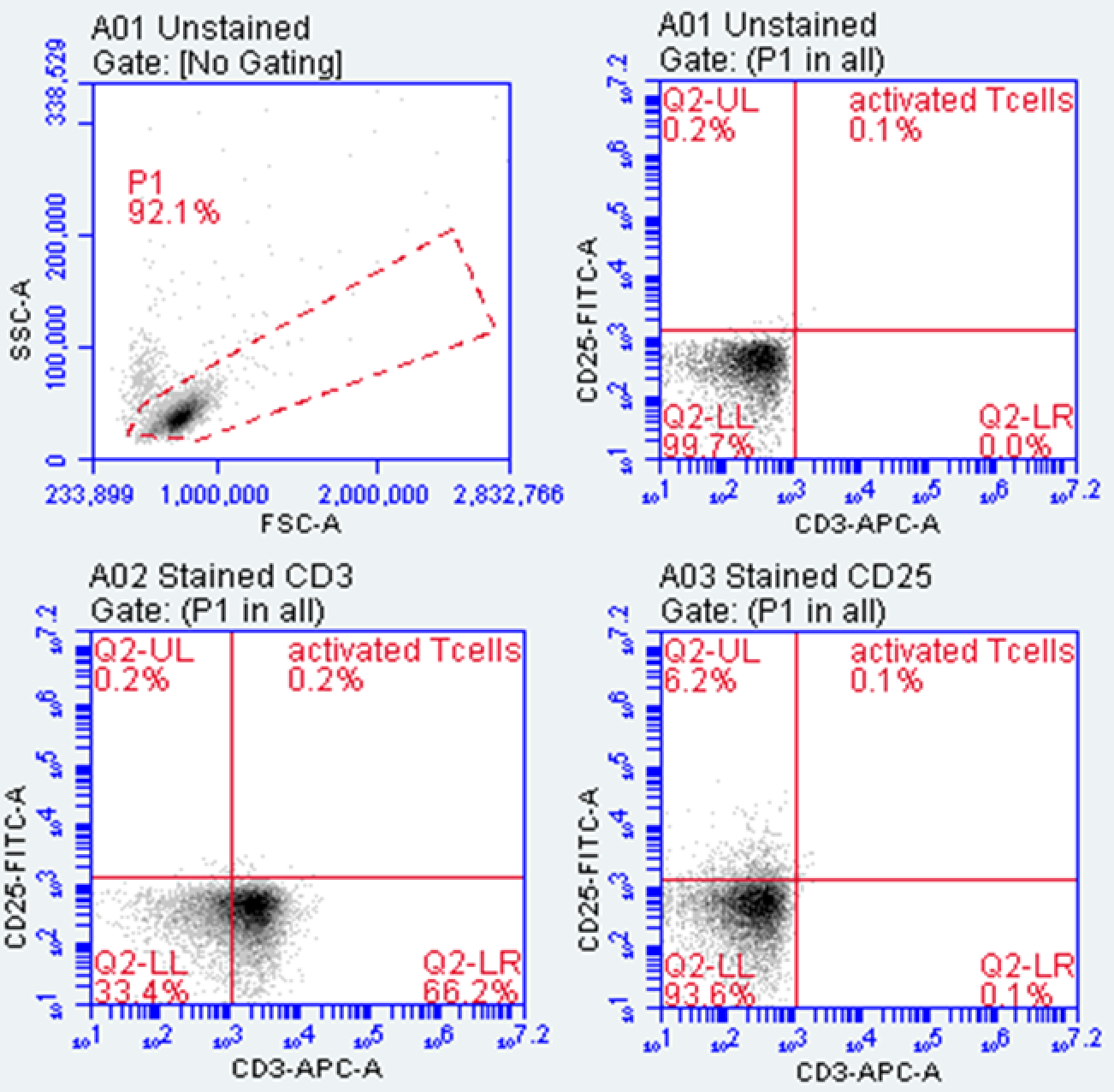

Supplement: Supplementary file 1 [file ijms-25-09423-s001.zip › PACE Corrected/Fig S1.tif]
